# One-pot selective synthesis of azoxy compounds and imines via the photoredox reaction of nitroaromatic compounds and amines in water

**DOI:** 10.1038/s41598-018-38100-6

**Published:** 2019-02-04

**Authors:** Hao Tan, XingChen Liu, JiHu Su, YingXiong Wang, XianMo Gu, DongJiang Yang, Eric R. Waclawik, HuaiYong Zhu, ZhanFeng Zheng

**Affiliations:** 1 State Key Laboratory of Coal Conversion, Institute of Coal Chemistry, Chinese Academy of Sciences, Taiyuan, 030001 China; 2 University of Chinese Academy of Sciences (UCAS), Beijing, 100049 China; 3 Department of Modern Physics, University of Science and Technology of China, Hefei, 230026 China; 4 School of Environmental Science and Engineering, Collaborative Innovation Center for Marine Biomass Fibers, Materials and Textiles of Shandong Province, Qingdao University, Qingdao, 266071 China; 5 School of Chemistry, Physics and Mechanical Engineering, Queensland University of Technology, Brisbane, QLD 4001 Australia

## Abstract

A facile one-pot two-stage photochemical synthesis of aromatic azoxy compounds and imines has been developed by coupling the selective reduction of nitroaromatic compounds with the selective oxidation of amines in an aqueous solution. In the first stage (light illumination, Ar atmosphere), the light excited nitroaromatic molecule abstract H from amine to form ArNO_2_H and amine radical, which then form nitrosoaromatic, hydroxylamine and imine compounds. Water acts as a green solvent for the dispersion of the reactants and facilitates the formation of nitrosoaromatic and hydroxylamine intermediate compounds. In the second stage (no light, air atmosphere), the condensation of nitrosoaromatic and hydroxylamine compounds yields aromatic azoxy product with the aid of molecular oxygen in air. This photochemical synthesis achieved both high conversion and high product selectivity (>99%) at room temperature.

## Introduction

The selective reduction of nitroaromatic compounds to azo/azoxy compounds and the selective oxidation of amines to imines are two important types of reactions. They are widely studied in the formation of molecules containing N=N or C=N bonds, which are important intermediates for the production of dyes and pharmaceuticals^1–3^. For the selective reduction of nitroaromatic compounds to azo and azoxy compounds, either reducing agent or catalytic routes are used. However, these processes normally generate hazardous by-produces or require harsh reaction conditions such as high temperature and pressure^4–6^. Recently, it has been reported that Au/ZrO_2_ and Cu/graphene can reduce nitroaromatic compounds to azo compounds <100 °C with high product selectivity (>99%) under incandescent light irradiation^7–9^. On the other hand, the selective oxidation of an amine to the corresponding imine is a extensively studied acid-catalyzed reaction^10^. The direct oxidation of amines using molecular oxygen under light irradiation has drawn a lot of attention lately, with metal oxides such as TiNb_2_O_7_^11^ and Nb_2_O_5_^12^ or metal free photocatalyst such as carbazolic conjugated microporous polymer^13^ exhibiting good catalytic activity for the oxidation reaction. In both of the photocatalytic selective reduction and oxidation reactions, the photocatalysts absorb incident light and transfer the light energy to the substrate via photo-induced carriers, which are low efficient processes because the photo-induced carriers are prone to recombination in the transmission process^14,15^.

Recently, catalyst-free photochemical reactions have shown great promise in providing efficient routes to prepare fine chemicals, e.g. the conversion of cyclohexane to adipic acid using UV light and ozone^16^. We questioned whether the selective reduction and oxidation steps can be coupled without the need of a catalyst if at least one of the substrates can directly absorb incident light to become activated. Given the light absorption of nitroaromatic compounds in the near-UV and the H-donating ability of amines^17^, it is possible that an amine can act as the reducing agent for the selective reduction of a nitroaromatic compound and itself is oxidized to its corresponding imine. If it works, both the reactants convert to valuable products without the need of a catalyst using a one-pot reaction. Evidently this process can reduce the use of reagents, simplify the separation steps and increase the yield^18^. Furthermore, the mechanism of this type of process will advance our fundamental knowledge of photochemical reaction kinetics. Herein, we design a facile photochemical “one-pot” approach for the synthesis of imine and azoxybenzene from the aqueous solution of amine and nitrobenzene without the addition of any catalyst. The light was then turned off and air was introduced into the reaction. As expected, the reaction system worked effectively with high selectivity. Importantly, the reaction was conducted in a green solvent - water.

## Results and Discussion

**Figure 1:**
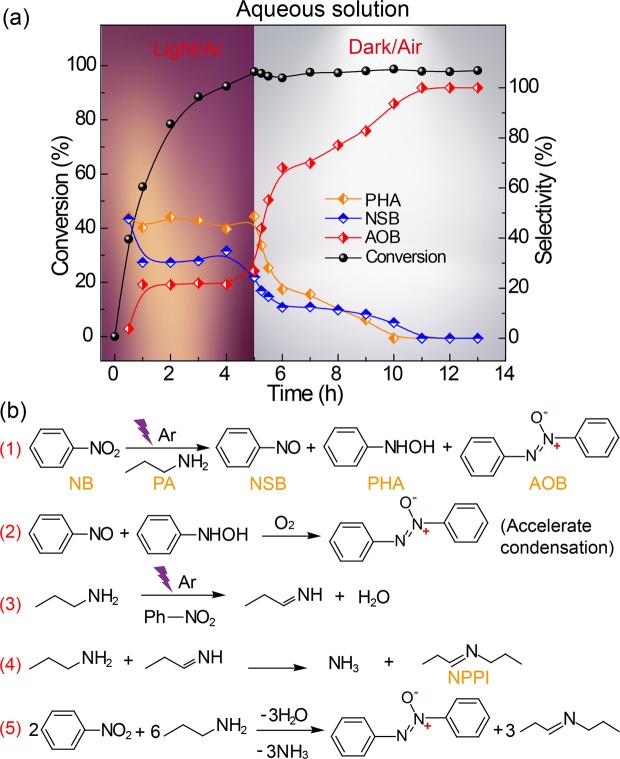
(**a**) The time profile of the changes in the reactants (NB and PA) and the products (PHA, NSB, and AOB) observed during the reaction, and (**b**) the main reaction equations summarized. Reaction conditions: 40 °C, 1 atm Ar, purple LED light, 1.5 mL of H_2_O, 0.5 mL of PA, 0.04 mmol of NB, 200 mW cm^−2^. (1), (2), (3), (4) and (5) in panel 1a represent the photoreaction, the air-aided condensation reaction, the formation process of propanimine and imine and the overall reaction, respectively.

**Figure 2:**
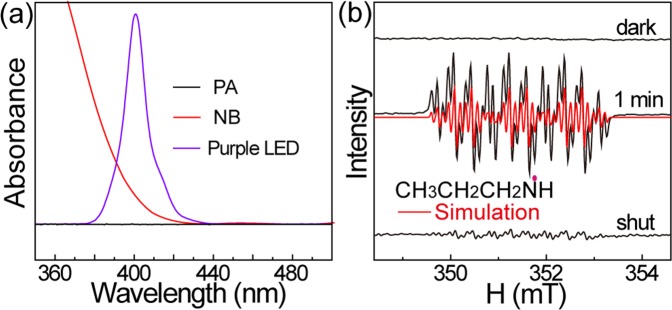
(**a**) The UV-Vis spectra of the reactants (PA and NB) and the output spectrum of the purple LED light. (**b**) The EPR spectra of the mixed solution of NB and PA in the presence and absence of light irradiation.

The reaction process between **nitrobenzene** (**NB**) and **propylamine** (**PA**) in aqueous solution can be divided into two main steps (Fig. 1a). The photoreaction process (1–5 h) and the exposure process (5–13 h). In the photoreaction process, more than 98% of NB can be converted at 5 h (the apparent quantum yield, AQY, is 0.25%, the calculation details are given in the ESI). Three main products from NB were detected with gas chromatography mass spectrometry (GC-MS) and high-performance liquid chromatography (HPLC) analysis, including **nitrosobenzene** (**NSB**), **phenyl-hydroxylamine** (**PHA**) and **azoxybenzene** (**AOB**). The proportion of the three products remained almost constant throughout the photoreaction process indicating that the reaction followed the equation Fig. 1b-1. In the exposure stage, the light was switched off and air was introduced into the reaction vessel. During the initial exposure stage (5–8 h), the condensation of PHA and NSB proceeded rapidly to yield AOB. The similar trend was observed when O_2_ instead of air was purged into the vessel. These imply that O_2_ in the air can promote the condensation reaction between NSB and PHA (Fig. 1b-2). It has been reported that the molecular oxygen in the air can destroy the nitrosobenzene radical anion formed during the reaction between NSB and PHA, which is favorable for the condensation reaction^19,20^. During the later stage (8–11 h), the PHA was not detected after 10 h, however, the NSB had disappeared until 11 h and the yield of AOB had increased from 92.6% to 98.2% when the reaction time was from 10 h to 11 h. It indicates there may exist a reaction pathway that can yield AOB from NSB, which is probably the rate-determining step. In a control experiment, PHA was detected by HPLC in the reaction between NSB and PA (Figure S1). When PA was replaced with toluene, AOB was not detected (Figure S2), indicating that PA plays an important role in converting NSB to AOB. To clarify whether PHA reacts in the presence of oxygen to yield AOB, pure PHA was exposed to air, and a mixture including NB, AOB and azobenzene was obtained. This indicates that AOB is yielded via the condensation between NSB and PHA and not via PHA reaction with O_2_. The oxidation product of PA, ***N*-propylpropanimine** (**NPPI**), was identified by nuclear magnetic resonance (NMR) spectroscopy (Figure S3 a–e). Based on the above results, a rational reaction equation (Fig. 1b-5) was conjectured. The Gibbs free energy of the reaction is −1.44 kJ mol^−1^ (Table S1), indicating that the reaction was thermodynamically spontaneous.

**Figure 3:**
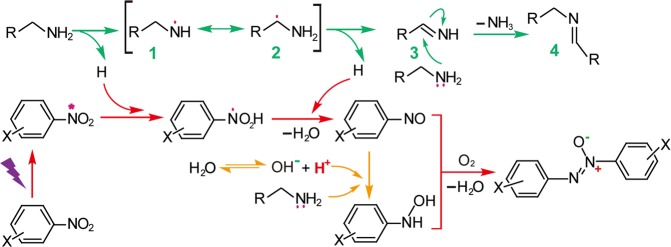
The proposed mechanism for the one-pot selective synthesis of aromatic azoxy compounds and imines.

To confirm whether this one-pot synthesis can be scaled up, the gram scale reaction was conducted by mixing 1.2 g of NB with 7.2 of PA (10–50 times greater than the previous photocatalytic studies^8,21^). As shown in Figure S4, conversion of 100% of NB is achieved to yield the NSB, PHA and AOB products under irradiation for 28 h. After the mixture were exposed at air for 120 h, only AOB and NPPI were observed besides solvent and the yield of AOB and NPPI was 99% and 38%, respectively (AQY = 0.23%). While the theoretical yield of NPPI should be 24% based on the equation Fig. 1b-5. This indicates that a condensation reaction between PA and propanimine [C_2_H_5_CH=NH, the direct oxidation product of PA attacked by the excited NB (Fig. 1b-3)] occurred, which contributed to the production of NPPI (Fig. 1b-4).

Neither AOB nor NPPI was achieved in the absence of light. The conversion rate of NB increased from 21% to 57% in aqueous solution [Figure S5a (red line)] and from 35% to 86% in pure PA solution [Figure S5b (red line)] when the light intensity was increased from 40 to 200 mW cm^−2^. These results indicate that the conversion of NB was driven by light. The conversion of NB was increased when the temperature was increased from 30 to 80 °C in aqueous solution while keeping other conditions identical (Figure S5a, blue line). However, in a pure PA or inert organic liquid solution, such as acetonitrile, toluene or cyclohexane (Figure S5b and c), the conversion rate remained constant with changing the reaction temperature. It leads us to further study the unique role of water. On one hand, the solubility of nitrobenzene is higher in an organic solvent than in an aqueous solution. A higher temperature can increase the solubility of nitrobenzene in H_2_O. On the other hand, the ionization of water is endothermic. Therefore, higher temperatures will promote the dissociation of water and produce more OH^−^ and H^+^ ions^22^ which will be beneficial to the reaction.

The hydrogen donating ability of ammonia, amine and water was investigated (Table S2). Trace amounts of NB were converted when using H_2_O (entry 1). In an NH_3_**·**H_2_O solution, 14% of NB was converted within 5 h (entry 2) and the main product was aniline, the over-reduced product. In a N_2_H_4_**·**H_2_O solution, the product selectivity was the same as that observed in NH_3_**·**H_2_O, however, the conversion was higher. When the –NH_2_ group is bonded to an alkyl group, such as methylamine or ethylamine, (entries 4 and 5), less than 5% of aniline and more AOB product were observed in the resulting products and the conversion rate was >97%. The alkyl group may affect the hydrogen donating ability of the –NH_2_ group, the reduction pathway of NB and the selectivity for AOB. Therefore, the reducing ability of the amines was in the order of:

$${{\rm{N}}}_{2}{{\rm{H}}}_{4}\,\cdot {{\rm{H}}}_{2}{\rm{O}} > {{\rm{NH}}}_{3}\,\cdot {{\rm{H}}}_{2}{\rm{O}} > {{\rm{CH}}}_{3}{{\rm{NH}}}_{2}\,\cdot {{\rm{H}}}_{2}{\rm{O}} > {{\rm{C}}}_{2}{{\rm{H}}}_{7}{{\rm{NH}}}_{2}\,\cdot {{\rm{H}}}_{2}{\rm{O}}$$

and H_2_O probably acts as a dispersant in the photoreaction process.

The substrate scope of this one-pot coupling reaction was explored (Table 1). Reactions using nitrobenzene derivatives bearing electron-donating groups (–CH_3_, –OCH_3_) and electron-withdrawing groups (–Cl, –CN) were studied (entries 1–4). The substrates reacted with PA to give corresponding azoxy compounds and NPPI with high selectivity. We also investigated the reactions of NB with a series of amines. When butylamine or benzylamine reacted with NB, the resulting products were the corresponding imine and AOB (entries 5 and 6). The yield of corresponding azoxy- and imines compounds are shown in Table S3.

**Table 1:** The photochemical reaction between various nitrobenzene derivatives (N.D.) and different amines.

Entry	Substrate		t_p_/t_d_ (h)^a^	Con._NB_ (%)^b^	Sel._AOB_ (%)^c^	Sel._imine_ (%)^d^
	N.D.	Amines				
1		C_3_H_7_NH_2_	5/48	99	>99	>99
2^e^		C_3_H_7_NH_2_	2/48	93	>99	>99
3^e^		C_3_H_7_NH_2_	2/48	97	>99	>99
4^e^		C_3_H_7_NH_2_	2/48	98	>99	>99
5		C_4_H_9_NH_2_	5/48	99	99	>99
6		PhCH_2_NH_2_	5/48	97	92	>99

^a^t_p_: the photochemical reaction time; t_d_: the time exposure to air in the absence of light. ^b^Conversion rate of nitro-compounds. ^c^Selectivity to azoxy-compounds. ^d^Selectivity to imines; ^e^Performed in a PA solution due to its poor solubility in an aqueous solution (2 mL of PA, 80 mW cm^−2^). Photochemical reaction conditions: deionized water (1.5 mL), amine (0.5 mL), nitro compound (0.04 mmol), 40 °C, 1 atm Ar, purple LED light (200 mW cm^−2^).

The absorption of light is the first step during a photochemical reaction. To understand how the coupled reaction was initiated, we compared the UV-Vis absorption spectra of the reactants and the output spectrum of the purple LED light (Fig. 2a). No absorption of PA was observed in the range >350 nm. The absorption of NB was in the wavelength range <440 nm, which was due to n-π* transitions of NB^23,24^. It is obviously that the light absorption of NB range overlaps with the light output spectrum of the purple LED in the 370–440 nm range. No conversion of NB was observed when using blue LED light (~460 nm) as the light source, which indicates that light with a length between 370 and 440 nm induces the conversion of NB.

A photochemical reaction normally involves radical formation pathway. The radicals formed during the reaction were determined by electron paramagnetic resonance (EPR) spectroscopy. No EPR signal was observed when the mixture of NB and PA was analyzed in the dark. After the mixture was irradiated under UV light for 1 min, an EPR signal with a complicated hyperfine feature was recorded *in situ* (Fig. 2b). The signal can be assigned to the nitrogen-centered PrNH• radical^25,26^. The results of the simulation show that the hyperfine constants from the coupled nitrogen and hydrogen in PrNH• are a_N_ = 11.6 G, a_H3_ = 3.6 G and a_H2_ = 1.2 G. The EPR signals observed for the PrNH• radicals are rather stable at room temperature under illumination. However, the signal intensity decayed quickly when the light was turned off. No EPR signal was observed from PA or NB alone in the dark or after 1 min of light irradiation (Figure S6). The results confirm that PrNH• radicals were generated only under light irradiation in the presence of NB. It has been reported that the n-π* transitions of NB can remove hydrogen atoms from an amine^17^. Therefore, it is sensible to infer that the purple light initiates the reaction between NB and PA by exciting the n-π* transitions of NB to produce radicals.

As stated earlier, NSB and PHA were gradually converted to AOB when exposed to air (Fig. 1). It has been reported that the reaction of NSB with an amine involves the initial nucleophilic attack of NSB by the alkylamine^27^, which affords an intermediate state containing a N–N bond such as **IM1** or **IM2** (Figure S7). Then, the elimination of PHA from the intermediate, which breaks the N–N bond, yields the final azoxy product^27,28^. However, according to our density functional theory (DFT) calculation results, no azoxy product but **FS1** and **FS2** generate through their reaction pathways. The DFT results show that a transition state (**TS1**) (Table S4), formed by the PA direct nucleophilic attack on NSB, is more feasible for the N–N bond cleavage reaction than the other two states. The energy barrier for this step is 372.4 kJ mol^−1^. **TS3** dissociates to give product **FS3**, which consists of a cation and an anion. The anion can readily react with H^+^ from the hydrolysis of water to form PHA via an energy barrier of −1455.3 kJ mol^−1^. In addition, the cation can react with the OH^−^ releasing one molecule of H_2_O to yield the RCH = NH product (Figure S8). The temperature can regulate the concentration of OH^−^ and H^+^ and affects the reaction, which indicates that water plays an important role in facilitating the reaction. PHA can condense with NSB with the assist of O_2_ to generate the azoxy product^19,20^ and releases 648.0 kJ mol^−1^ of energy.

It is noted that the selectivities to AOB and NPPI were very high and no further conversion was observed even after long time reaction (28 h). This could be due to that N=N bond (length of 1.24 Å) is stronger than N-O bond (length of 1.29 Å)^29^ and the reduction effect of amine is insufficient for further reduction in our condition. The wavelength of purple light also contributes to the high selectivity^17^. Aniline, an over-reduction product, was detected when Xenon lamp (full solar spectrum) was the light source (Figure S9).

Based on the above analysis, a reaction mechanism is proposed (Fig. 3). Firstly, the nitroaromatic compound was irradiated to form the excited states that abstract hydrogen from the amine forming the PhNO_2_H radical^24,30^ and an amine radical **(1)**. The latter was subsequently converted to a carbon radical **(2)** by means of a rearrangement and the radical loses a hydrogen atom to form RCH = NH **(3)**. Then, the lone pair of electrons in a second amine molecule participates in the nucleophilic attack of RCH = NH and eliminates an ammonia to form the final coupled imine product **(4)**^31^. The PhNO_2_H radical further give NSB via an PhN(OH)_2_ intermediate. Under the synergistic effect of water and amines, NSB can be converted to PHA. Finally, NSB reacts with PHA and yields AOB through a condensation reaction, where O_2_ molecules in the air facilitate the process.

## Conclusions

In conclusion, the photochemical protocol reported in the present study provides a facile and effective route for the preparation of high value azoxy compounds and imines. It reveals that the photochemical properties of nitroaromatic compounds, including their excitation by light and the ability of the light excited molecules to generate radicals, can be utilized for synthesis of fine chemicals under mild conditions with high selectivity if the reaction conditions are well controlled (e.g. light wavelength, solvent, atmosphere). These photochemical properties should also be taken into account in studies of photocatalytic reactions involving nitroaromatic compounds.

## Methods

### Photoreaction test

In general, all chemicals were used as received without further purification. The reduction of nitrobenzene was conducted under an Ar atmosphere (1 atm) in a 10 mL (25 mL for gram scale) round-bottomed pyrex glass flask with a sealed spigot and a magnetic stirrer. A purple LED lamp (~400 nm) was used as the light source. In the reaction, the concentration of nitrobenzene was 0.02 mol/L, unless specified otherwise, dissolved in an aqueous solution of PA at a volume ratio of 3:1. The concentration of nitrobenzene was increased 50–fold for the large-scale experiment. The product compositions were analyzed and determined by means of an Agilent HP5973 mass spectrometer and Agilent 1260 high efficiency liquid chromatography. The Agilent Series are equipped with a vacuum degasser, a quaternary pump, an autosampler and a DAD system, connected to a Agilent ChemStation software. A C18 column (250 × 4.6 mm i.d., 5 μm) were used. Flow rate was 1 mL min-1. Detection wavelength was at 280 nm. Solvents that constituted the mobile phase were (A) methanol and (B) water with the volume ratio 50:50.

#### N-propylpropanimine (NPPI)

1.2 g nitrobenzene and 7.2 g propylamine was mixed. After the reaction (the total weight is about 8 g), the mixture was distilled at 85 °C to separate the imine/propylamine and azo compounds. About 5 g of imine and propylamine transparent mixture was collected: ^1^H NMR (400 MHz, CDCl_3_, 25 °C, TMS) δ = 0.88 (t, *J* = 7.4 Hz, 2 H); δ = 1.09 (td, *J* = 7.6, 1.2 Hz, 3 H); δ = 1.61 (qd, *J* = 7.2, 1.2 Hz, 2 H); δ = 2.32–2.19 (m, 1 H); δ = 3.32 (tt, *J* = 7.0, 1.2 Hz, 2 H); δ = 7.64 (td, *J* = 4.6, 1.3 Hz, 1 H). ^13^C NMR spectrum of imine (100 MHz, CDCl_3_): δ 10.25, 11.57, 23.76, 28.90, 63.01, 165.62.

### Characterization

EPR *in situ* photochemical experiments: The formation of paramagnetic intermediates upon irradiation of solutions was monitored *in situ* using an EPR X-band spectrometer (Bruker Emxplus-10/12) at RT. The irradiation source was a 100 W Hg Arc lamp power supply (LOT, LSN161). Typical EPR spectrometer settings in a standard photochemical experiment were: microwave power, 10.02 Mw; microwave frequency, ca 9.863 GHz; center field, 348.0 mT; sweep width, 50 mT; scan, 60 s; time constant, 0.01 ms; modulation amplitude, 0.6 mT. In the water added measurement, 1 mL of nitrobenzene mixed with 100 uL deionized water and ultrasonic for 5 min.

Diffuse-reflectance UV-vis spectra were measured on an UV-vis spectrophotometer (Shimadzu UV-3600 -spectrometer). NMR spectra were acquired on a Bruker AV-III 400 MHz NMR spectrometer (9.39 T) equipped with a 5 mm PABBO BB/19F-1H/D Z-GRD probe and autosampler at room temperature. ^1^H and ^13^C NMR were obtained at frequencies of 400.13 MHz and 100.61 MHz respectively. The chemical shifts for ^1^H NMR and ^13^C NMR were referenced of the residual protons of CDCl_3_ (7.262 for ^1^H, 77.01 for ^13^C). When peak multiplicities were reported, the following abbreviations were used: s-singlet, d-doublet, t-triplet, q-quartet, m-multiplet, br-broadened. The pulse programs for COSY and HSQC acquisition are “cosygpppqf” and “hsqcetgpsi2”, respectively. The collected 1D and 2D NMR data were processed using Bruker Topspin 3.1 software.

## Supplementary information

Supplementary Information
