# Supplementary material for: One-pot selective synthesis of azoxy compounds and imines via the photoredox reaction of nitroaromatic compounds and amines in water
Source: Sci Rep. 2019 Feb 4;9:1280. doi: 10.1038/s41598-018-38100-6 (PMC6361970; doi:10.1038/s41598-018-38100-6)
Supplement: Supplementary file 1 — Supplementary Information [file 41598_2018_38100_MOESM1_ESM.docx]

Supplementary Information

One-pot selective synthesis of azoxy compounds and imines via the photoredox reaction of nitroaromatic compounds and amines in water

Hao Tan,^1,2^ XingChen Liu,^1^ JiHu Su,^3^ YingXiong Wang,^1^ XianMo Gu^1,*^ DongJiang Yang,^4^ Eric R. Waclawik,^5^ HuaiYong Zhu,^5^ and ZhanFeng Zheng^1,*^

^1^State Key Laboratory of Coal Conversion, Institute of Coal Chemistry, Chinese Academy of Sciences, Taiyuan 030001, China.

^2^University of Chinese Academy of Sciences (UCAS), Beijing, 100049, China.

^3^Department of Modern Physics, University of Science and Technology of China, Hefei 230026, China.

^4^School of Environmental Science and Engineering, Collaborative Innovation Center for Marine Biomass Fibers, Materials and Textiles of Shandong Province, Qingdao University, Qingdao 266071, China.

^5^School of Chemistry, Physics and Mechanical Engineering, Queensland University of Technology, Brisbane, QLD 4001, Australia.

**Contents**

Experimental Section

Table S1 - Gibbs Free Energy of all substrate

Table S2 - The photochemical reaction between nitrobenzene and different hydrogen sources in an aqueous solution

Table S3 - The photochemical reaction between various nitrobenzene derivatives and different amines.

Table S4 - The Cartesian coordinates of the transition state structures in xyz format

Figure S1 - The HPLC spectrum of the reaction liquid

Figure S2 - The intermediate reaction results for this reaction system

Figure S3 - The ^1^H NMR, ^13^C NMR, ^13^C-dept, 2D ^1^H-^1^H COSY and 2D ^1^H-^13^C me-HSQC spectrum of imine

Figure S4 - The time profile of the changes in the reactants and the products observed during the reaction

Figure S5 - Influence of light intensity and temperature on the conversion of nitrobenzene

Figure S6 - The EPR spectra of PA, NB in the presence and absence of light irradiation

Figure S7 - The free energy profiles for the reaction of NSB with PA

Figure S8 - The probable mechanism profiles of the generation process of PHA and propanimine

Figure S9 - The spectrum of Xenon lamp, LED purple and blue light

**Experimental Section**

Materials

Nitrobenzene and nitrobenzene derivatives (-CH_3_, -OCH_3_, -Cl, -F), ammonium hydroxide solution (28-30%), hydrazine hydrate (85%), methylamine solution (25-30%), ethylamine solution (65-70%), propylamine, benzylamine, phenethylamine, nitrosobenzene, acetonitrile, toluene and cyclohexane were purchased from Sinopharm Chemical Reagent Co., Ltd, China. Phenylhydroxylamine, azoxybenzene and azobenzene were purchased from aladdin. All chemicals were used as received without further purification.

**Apparent quantum yield**

In the gram scale reaction, reaction conditions: 10 mL of PA, 10 mmol of NB, 1 atm Ar. A 400 nm UV-LED light was used, light intensity was 160 mWcm^-2^, energy can be obtained on the surface (8.0 cm^2^) of the reaction solution. 99.2% of NB was consumed after 28 h.

$$apparent quantum yield \left( \% \right)=\frac{0.01 mol\times0.992}{\frac{E}{\mathrm{hv}}}=\frac{0.01 mol\times0.992hc}{E\lambda}=\frac{6.02\times{10}^{23}\times0.992\times0.01\times6.63\times{10}^{-34}\times3\times{10}^{8}}{160\times8\times{10}^{-3}\times28\times3600\times400\times{10}^{-9}}=0.23\%$$

In the typical reaction, reaction conditions: 1.5 mL of H_2_O, 0.5 mL of PA, 0.04 mmol of NB, 1 atm Ar. A 400 nm UV-LED light was used, light intensity was 200 mWcm^-2^, energy can be obtained on the surface (1.3 cm^2^) of the reaction solution. 98.3% of NB was consumed after 5 h.

$$apparent quantum yield \left( \% \right)=\frac{0.04 mmol\times0.997}{\frac{E}{\mathrm{hv}}}=\frac{0.04 mmol\times0.983hc}{E\lambda}=\frac{6.02\times{10}^{23}\times0.983\times0.04\times{10}^{-3}\times6.63\times{10}^{-34}\times3\times{10}^{8}}{200\times1.3\times{10}^{-3}\times5\times3600\times400\times{10}^{-9}}=0.25\%$$

**Computational Method**

All calculations were performed within the framework of density functional theory (DFT) at B3LYP level of theory with the Gaussian 09 package[^1^](#_ENREF_1). All atoms were treated with the 6-311++g** basis set. The geometries of the molecules were optimized to potential energy minimum without any geometrical constraint, and were verified with frequency calculations. The transition states were also verified with frequency calculations, followed by intrinsic reaction coordinate (IRC) calculations to trace the reaction paths. The free energies (at 298.15 K) were calculated using the standard statistical thermodynamic method in Gaussian via partition function, with contributions from electronic, translational, rotational, and vibrational degrees of freedom.[^2^](#_ENREF_2)


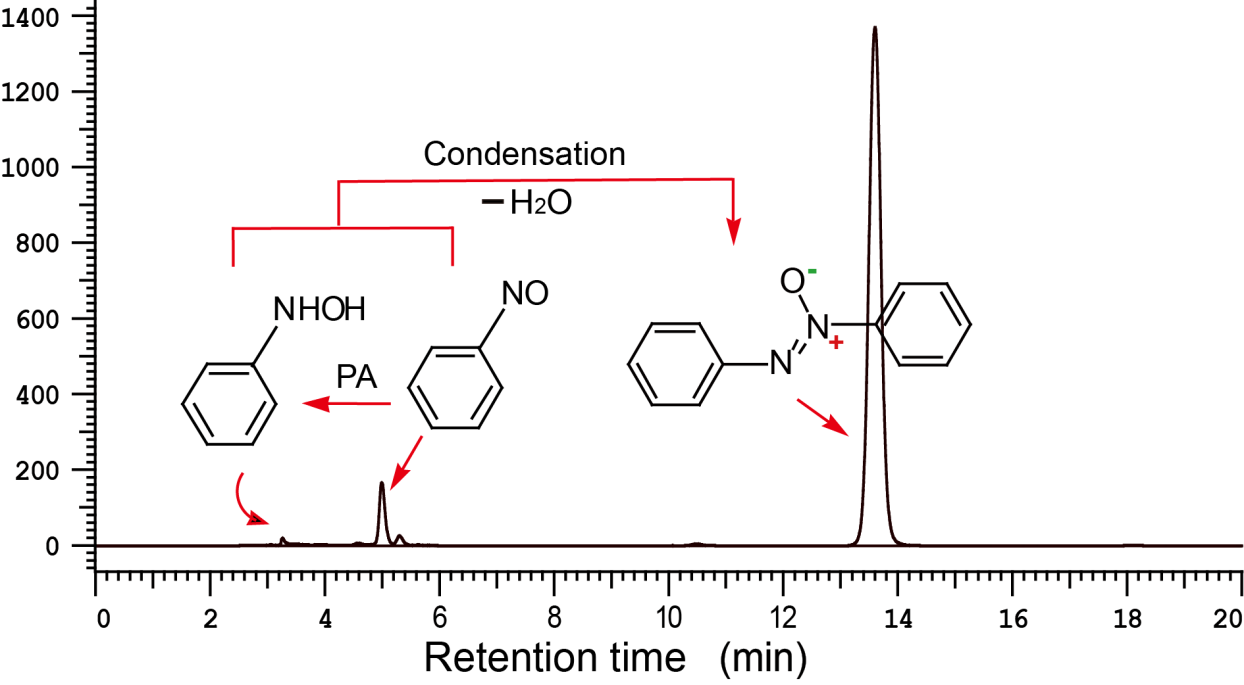


**Figure S1.** The HPLC spectrum of the reaction liquid


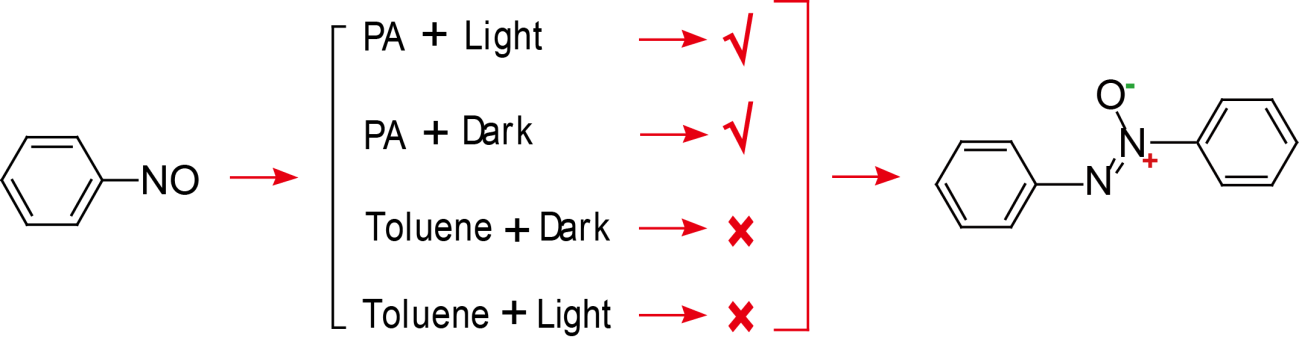


**Figure S2.** The intermediate reaction results for this reaction system. Reaction condition: Purple Light, t = 1 h, 2 mL of PA or toluene, 0.04 mmol of nitrosobenzene, room temperature, 1 atm air.


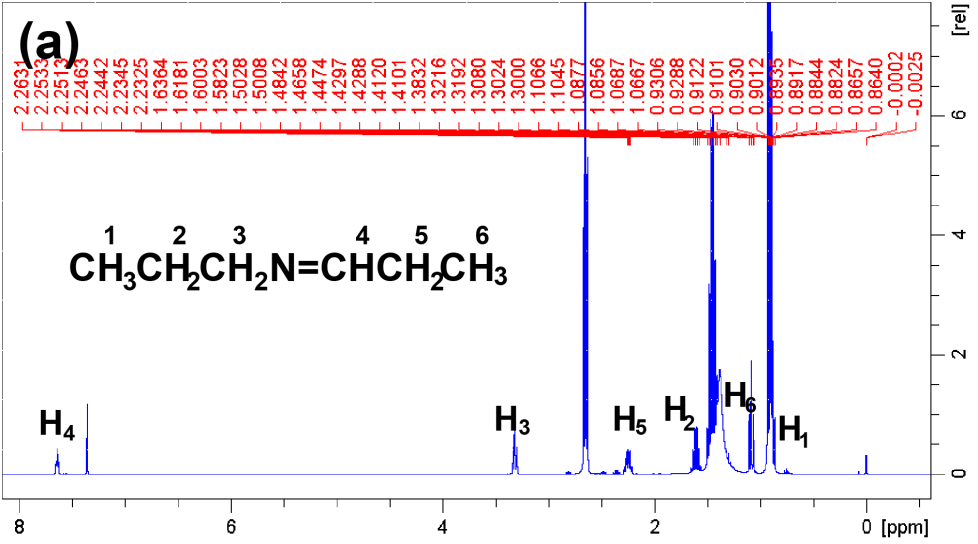


Figure S3a. The ^1^H NMR spectrum of imine (CDCl_3_, 400 MHz, ppm): δ = 0.88 (t, *J* = 7.4 Hz, 2H); δ = 1.09 (td, *J* = 7.6, 1.2 Hz, 3H); δ = 1.61 (qd, *J* = 7.2, 1.2 Hz, 2H); δ = 2.32 - 2.19 (m, 1H); δ = 3.32 (tt, *J* = 7.0, 1.2 Hz, 2H); δ = 7.64 (td, *J* = 4.6, 1.3 Hz, 1H).


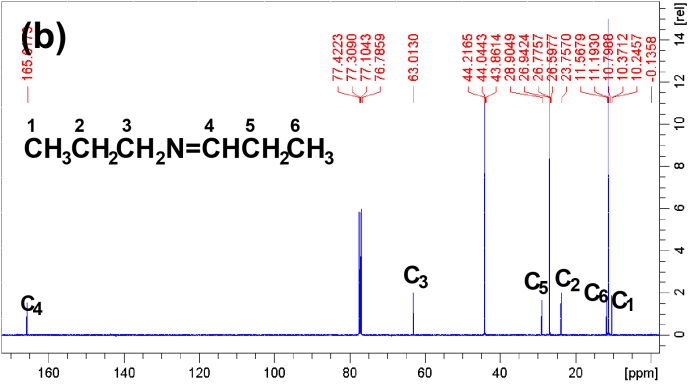


Figure S3b. The ^13^C NMR spectrum of imine (CDCl_3_, 100 MHz, ppm): 1C (δ = 10.25); 2C (δ = 23.76); 3C (δ = 63.01); 4C (δ = 165.62); 5C (δ = 28.90); 6C (δ = 11.57).


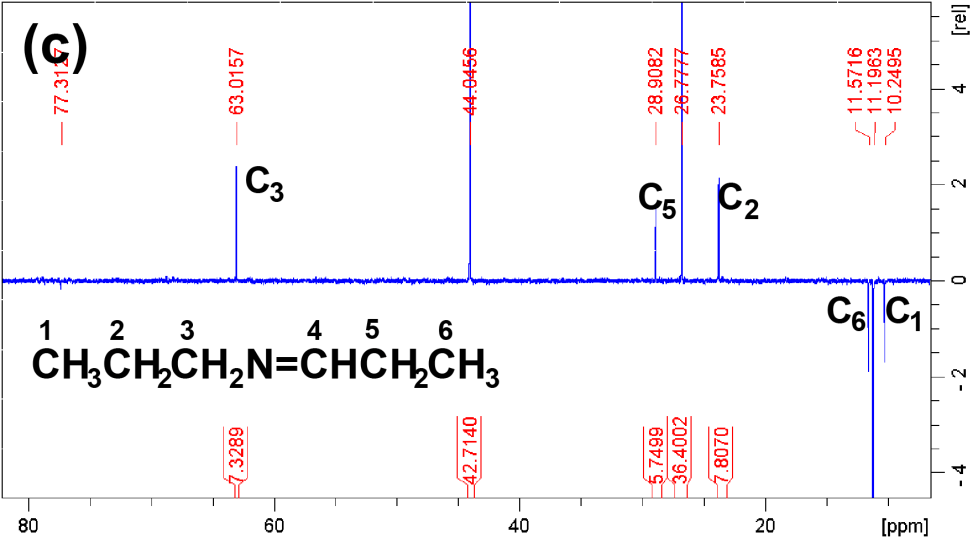


Figure S3c. The ^13^C-dept NMR spectrum of imine (135°). CH and CH_3_ are upward, CH_2_ is down, quaternary carbon cannot be detected.


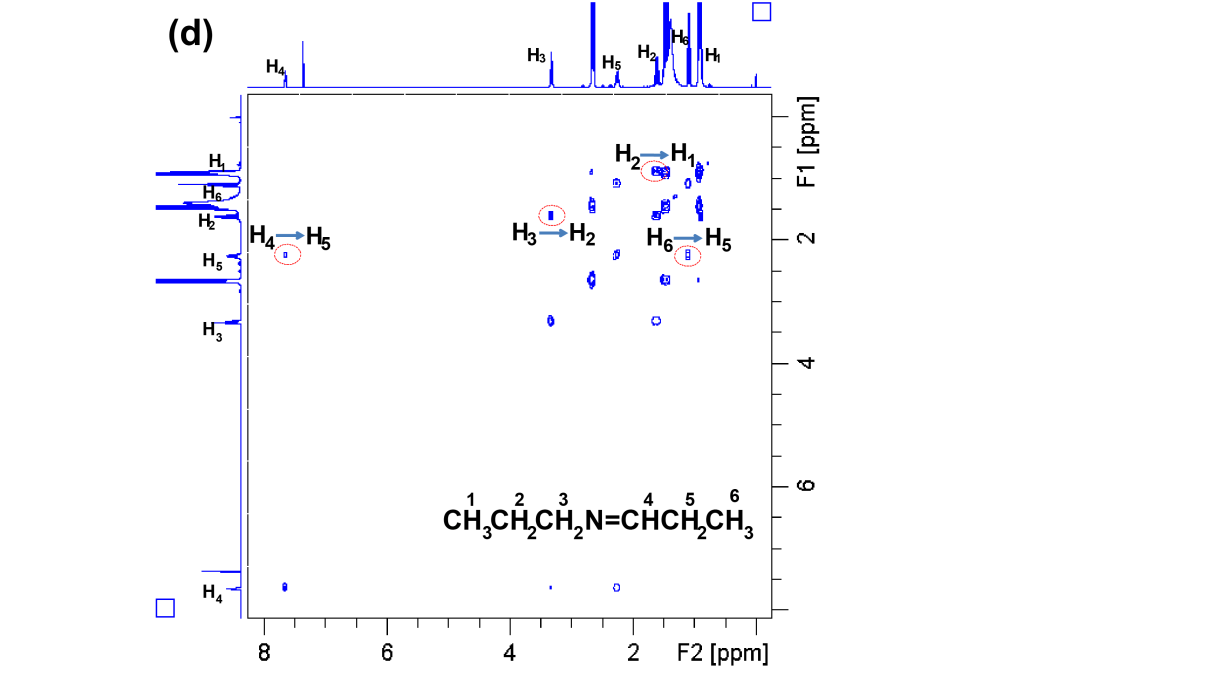


Figure S3d. The 2D ^1^H-^1^H COSY NMR spectrum of imine


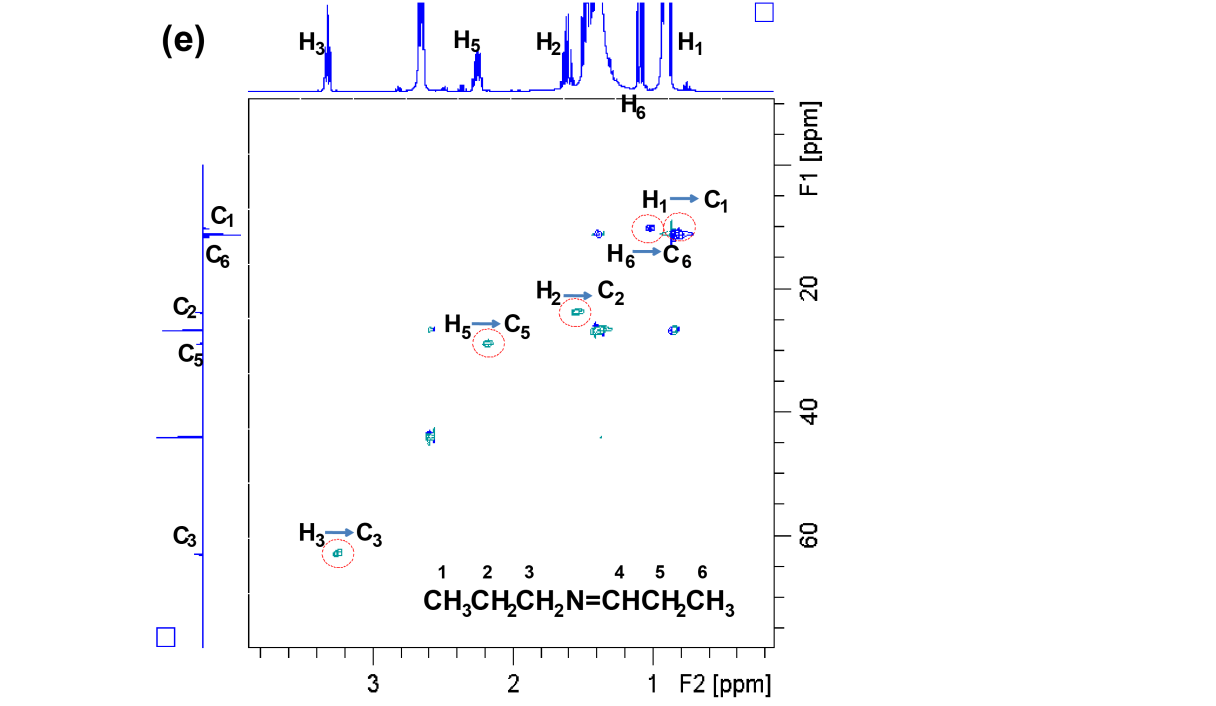


**Figure S3e.** The 2D ^1^H-^13^C me-HSQC NMR spectrum of imine

**Table S1:** Gibbs Free Energy of all substrate.

|  | **Substrate** | **Gibbs Free Energy**  **(kJmol^-1^)** |
| --- | --- | --- |
| **a** | Nitrobenzene | -436.80 |
| **b** | Propylamine | -174.45 |
| **c** | Water | -76.46 |
| **d** | Ammonia | -56.57 |
| **e** | Azoxybenzene | -647.96 |
| **f** | Imine | -291.15 |
| **△G = 3(f + d + c) + e - 6b - 2a** | | |
| **△G = -1.44 kJmol^-1^** | | |


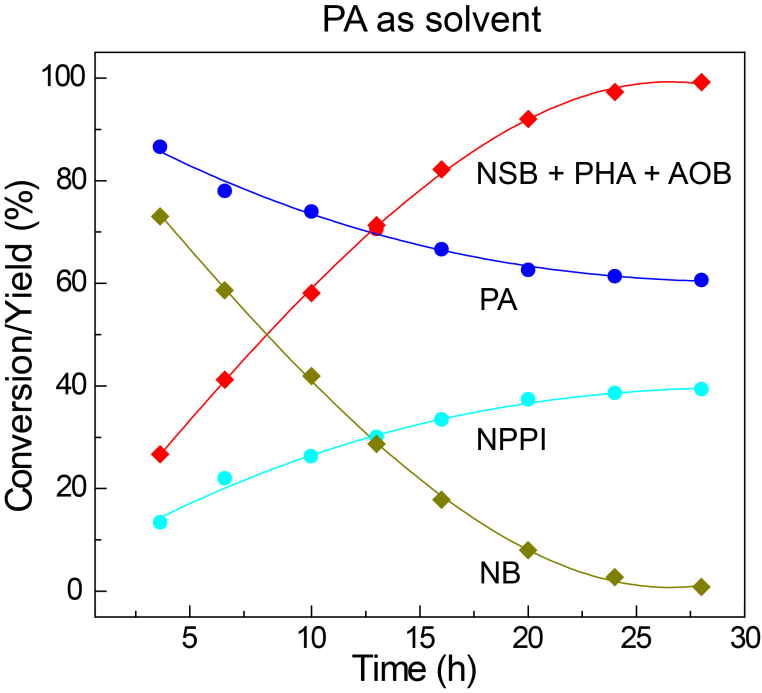


Figure S4. The time profile of the changes in the reactants (NB and PA) and the products [PHA, NSB, AOB and N-propylpropanimine (NPPI)] observed during the reaction. Reaction conditions: 40 °C, 1 atm Ar, purple LED light (400 nm), 7.2 g PA, 1.2 g NB, 160 mWcm^-2^.


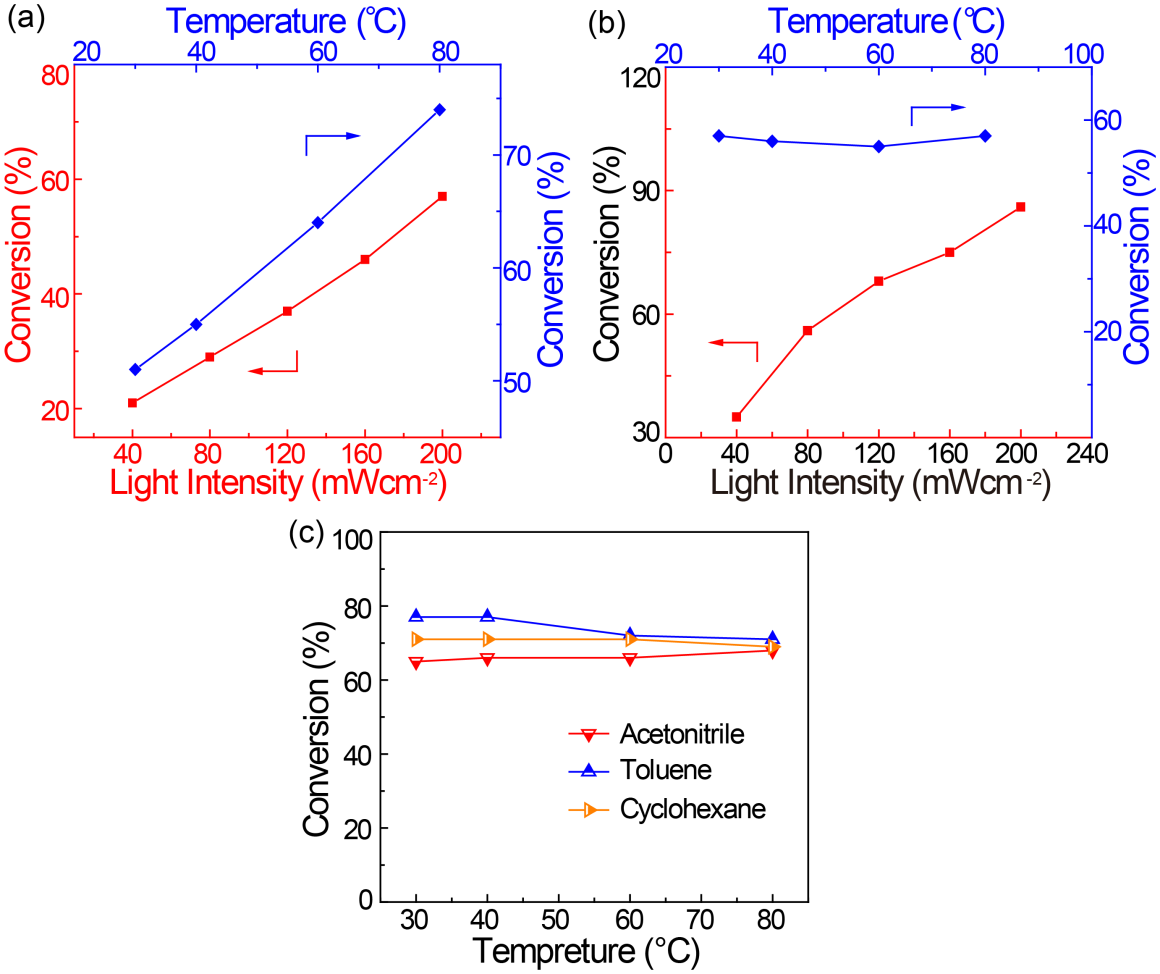


**Figure S5.** Influence of light intensity and temperature on the conversion of nitrobenzene. Reaction conditions: 0.04 mmol of NB, 1 atm Ar, purple LED light (400 nm). (a) 1.5 mL of H_2_O, 0.5 mL of PA, 0.04 mmol of NB. (b) 80 mWcm^-2^ irradiation, pure PA solution. (c) 200 mWcm^-2^ irradiation, inert organic liquid (acetonitrile, toluene and cyclohexane), the volume ratio of inert organic liquid and propylamine was 3:1. The mixture was exposed to air for 8 h after the photochemical reaction.

**Table S2:** The photochemical reaction between nitrobenzene and different hydrogen sources in an aqueous solution.

| **Entry** | **Solution** | **Con._NB_** | **Sel._Aniline_** | **Sel._PHA_** | **Sel._NSB_** | **Sel._AOB_** |
| --- | --- | --- | --- | --- | --- | --- |
| 1 | H_2_O^[a]^ | --^[f]^ | -- | -- | -- | -- |
| 2 | NH_3_·H_2_O^[b]^ | 14 | 50 | 35 | 6 | 9 |
| 3 | N_2_H_4_·H_2_O^[c]^ | 98 | 28 | 72 | -- | -- |
| 4 | CH_3_NH_2_·H_2_O^[d]^ | 97 | 3 | 23 | 61 | 13 |
| 5 | C_2_H_5_NH_2_·H_2_O^[e]^ | >99 | 2 | 42 | 10 | 46 |

[a] 2 mL of deionized water. [b] 2 mL of 28%–30% ammonium hydroxide solution. [c] 1.3 mL of deionized water and 0.7 mL of 85% hydrazine hydrate. [d] 2 mL of 25-30% methylamine solution. [e] 1 mL of deionized water and 1 mL of 65-70% ethylamine solution. [f] No content. The other reaction conditions were kept identical: NB (0.04 mmol), 40 °C, 1 atm Ar, purple LED light (400 nm), 200 mWcm^−2^.

**Table S3** The photochemical reaction between various nitrobenzene derivatives (N.D.) and different amines.

| Entry | | Substrate | | | t_p_/t_d_  (h)^a^ | | Con._NB_ (%)^b^ | Yield._AOB_  (%)^c^ | Yield._imine_  (%)^d^ |
| --- | --- | --- | --- | --- | --- | --- | --- | --- | --- |
|  |  | N.D. | Amines | |  |  |  |  |  |
| 1 |  | | | C_3_H_7_NH_2_ | | 5/48 | 99 | 99 | 2.0 |
| 2^e^ |  | | | C_3_H_7_NH_2_ | | 2/48 | 93 | 93 | 2.0 |
| 3^e^ |  | | | C_3_H_7_NH_2_ | | 2/48 | 97 | 97 | 2.0 |
| 4^e^ |  | | | C_3_H_7_NH_2_ | | 2/48 | 98 | 98 | 0.5 |
| 5 |  | | | C_4_H_9_NH_2_ | | 5/48 | 99 | 98 | 2.4 |
| 6 |  | | | PhCH_2_NH_2_ | | 5/48 | 97 | 89 | 2.1 |
| ^a^ t_p_: the photochemical reaction time; t_d_: the time exposure to air in the absence of light. ^b^ Conversion rate of nitro-compounds. ^c^ Yield to azoxy-compounds. ^d^ Yield to imines; ^e^ Performed in a PA solution due to its poor solubility in an aqueous solution (2 mL of PA, 80 mWcm^−2^). Photochemical reaction conditions: deionized water (1.5 mL), amine (0.5 mL), nitro compound (0.04 mmol), 40 °C, 1 atm Ar, purple LED light (200 mWcm^−2^). | | | | | | | | | |


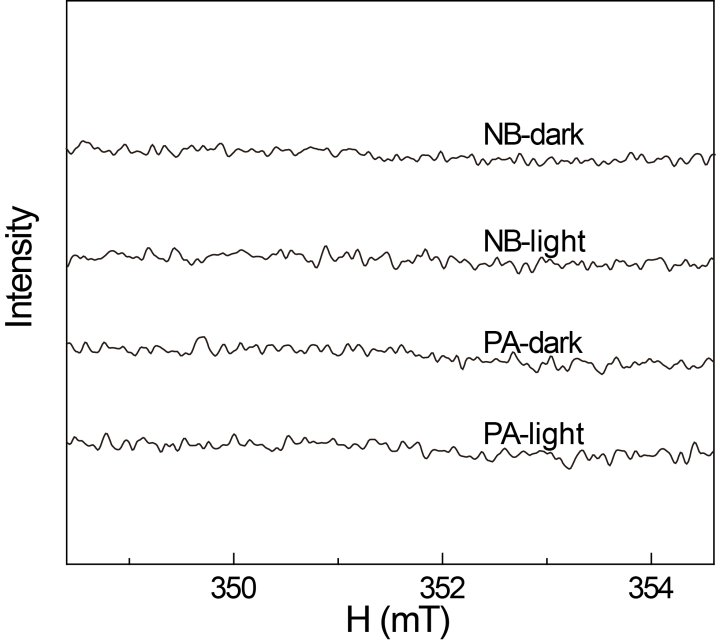


**Figure S6.** The EPR spectra of PA, NB in the presence and absence of light irradiation.


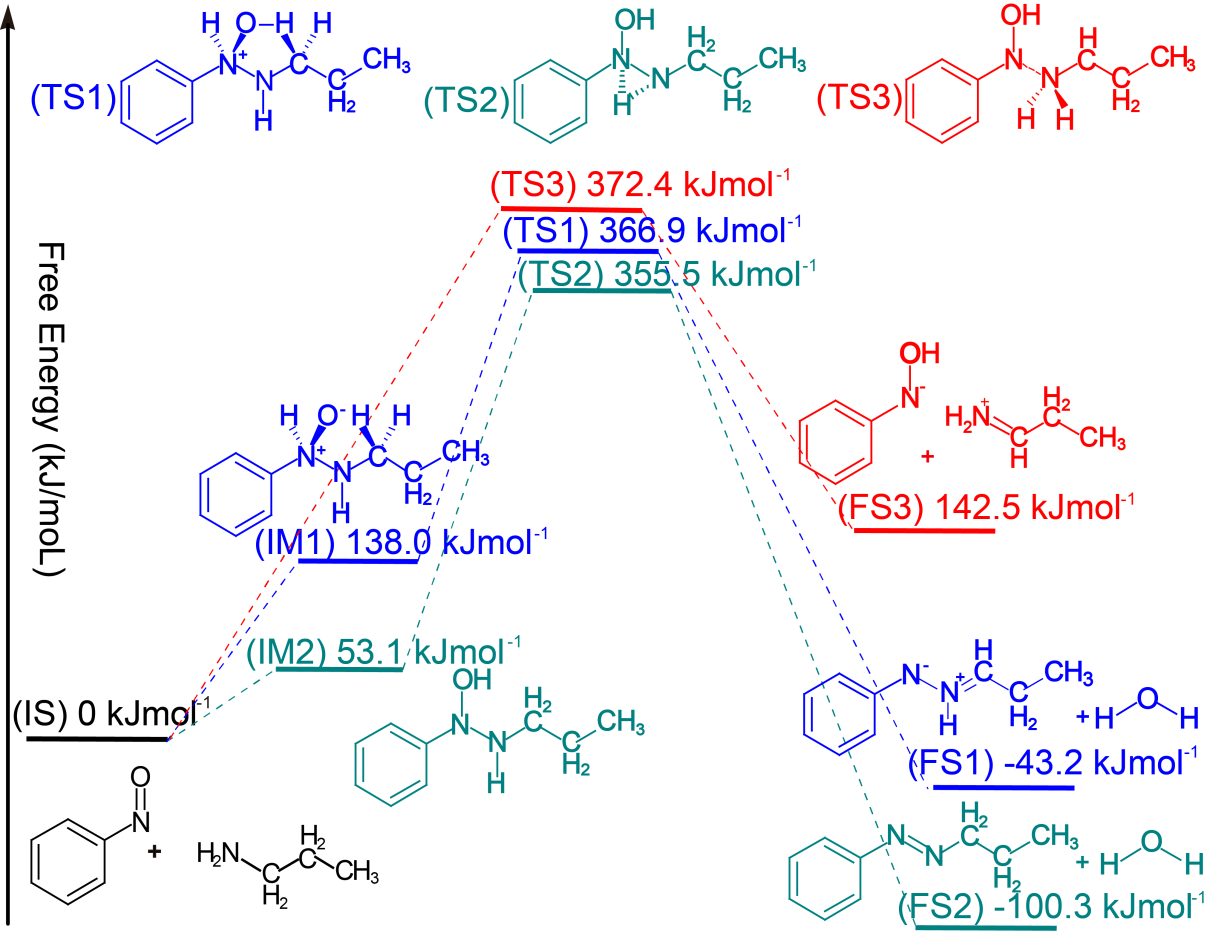


**Figure S7.** The free energy profiles for the reaction of NSB with PA. The Cope-type elimination (cyan), the Saytzeff type E2 elimination (blue) and the imine elimination (red) routes are distinguished using different colors. **IS** : initial state; **IM** : intermediate state; **TS** : transition state; **FS** : final stste.

Lamson et al.,[^3^](#_ENREF_3) have suggested that the reaction proceeds via a Cope-type elimination, which goes through a five-membered ring state **TS1**. Wu et al.[^4^](#_ENREF_4) however, argued that it is a Saytzeff type E2 elimination reaction and involves transition state **TS2** (the Cartesian co-ordinates axis is shown in Table S3). Combined with literature and our calculation result, for the Cope-type elimination, intermediate **IM1** is formed in the first step, which then undergoes a five-membered ring transition state **TS1** to give **FS1**. In the Saytzeff type E2 elimination pathway, the intermediate state **IM2** formed first, then it yields **FS2** via transition state **TS2** where a bridged H atom is shared by two N atoms. It crosses a barrier of 302.4 kJmol^-1^ from **IM2** to **TS2**. Once compound **FS1** or **FS2** is formed, the reaction terminates and no PHA and azoxy compound can be formed.

**Table S4:** The Cartesian coordinates of the transition state structures in xyz format

***Structure TS1***

| **Atom** | **x** | **y** | **z** | **Atom** | **x** | **y** | **z** |
| --- | --- | --- | --- | --- | --- | --- | --- |
| C | 1.03303 | 0.32417 | -0.21528 | C | 2.32476 | 0.68588 | 0.17232 |
| C | 0.71206 | -1.01888 | -0.44522 | C | 1.68027 | -1.99796 | -0.24975 |
| C | 3.28768 | -0.30749 | 0.34636 | C | 2.97213 | -1.64813 | 0.14367 |
| N | 0.00130 | 1.30310 | -0.45480 | N | -1.16761 | 1.06625 | 0.45606 |
| C | -2.42367 | 0.55905 | -0.22814 | H | -1.30647 | 1.99200 | 0.86591 |
| O | 0.47108 | 2.60603 | -0.13631 | C | -2.98507 | -0.57840 | 0.60609 |
| C | -4.04869 | -1.35603 | -0.17557 | H | 2.56912 | 1.72702 | 0.33049 |
| H | -0.28381 | -1.28351 | -0.77844 | H | 1.42538 | -3.03724 | -0.42322 |
| H | 3.72717 | -2.41353 | 0.28252 | H | 4.29119 | -0.02342 | 0.64722 |
| H | -4.48267 | -2.15789 | 0.42893 | H | -4.85407 | -0.68671 | -0.49019 |
| H | -3.62296 | -1.80763 | -1.07741 | H | -2.08167 | 0.21172 | -1.20479 |
| H | -2.20570 | -1.29960 | 0.95499 | H | -3.45470 | -0.20136 | 1.52544 |
| H | 0.19941 | 3.12429 | -0.90580 | H | -0.85672 | 0.44513 | 1.23697 |

***Structure TS3***

| **Atom** | **x** | **y** | **z** | **Atom** | **x** | **y** | **z** |
| --- | --- | --- | --- | --- | --- | --- | --- |
| N | 0.13798 | 0.65904 | -0.22274 | O | 0.21455 | 1.87968 | 0.62029 |
| C | -1.23854 | 0.17780 | -0.10701 | C | -1.47097 | -1.16490 | 0.17424 |
| C | -2.28747 | 1.06982 | -0.31153 | C | -2.78916 | -1.61834 | 0.23576 |
| C | -3.59568 | 0.60481 | -0.23978 | C | -3.85032 | -0.74114 | 0.03072 |
| H | 0.98534 | 2.34601 | 0.27106 | N | 1.16460 | -0.41481 | 0.00508 |
| C | 2.47387 | 0.23142 | 0.04842 | C | 3.53994 | -0.85053 | -0.16426 |
| C | 4.96838 | -0.31502 | -0.03495 | H | -2.07852 | 2.11282 | -0.50983 |
| H | -0.62393 | -1.81625 | 0.33301 | H | -2.98152 | -2.66354 | 0.44982 |
| H | -4.41753 | 1.29425 | -0.39531 | H | -4.87187 | -1.10048 | 0.08119 |
| H | 2.63497 | 1.02307 | -0.72489 | H | 2.65575 | 0.71253 | 1.02357 |
| H | 3.36967 | -1.64544 | 0.56839 | H | 3.39295 | -1.30128 | -1.15108 |
| H | 5.17376 | 0.46489 | -0.77541 | H | 5.14546 | 0.11341 | 0.95649 |
| H | 5.70077 | -1.11255 | -0.18517 | H | 0.50477 | 0.64402 | -1.17813 |


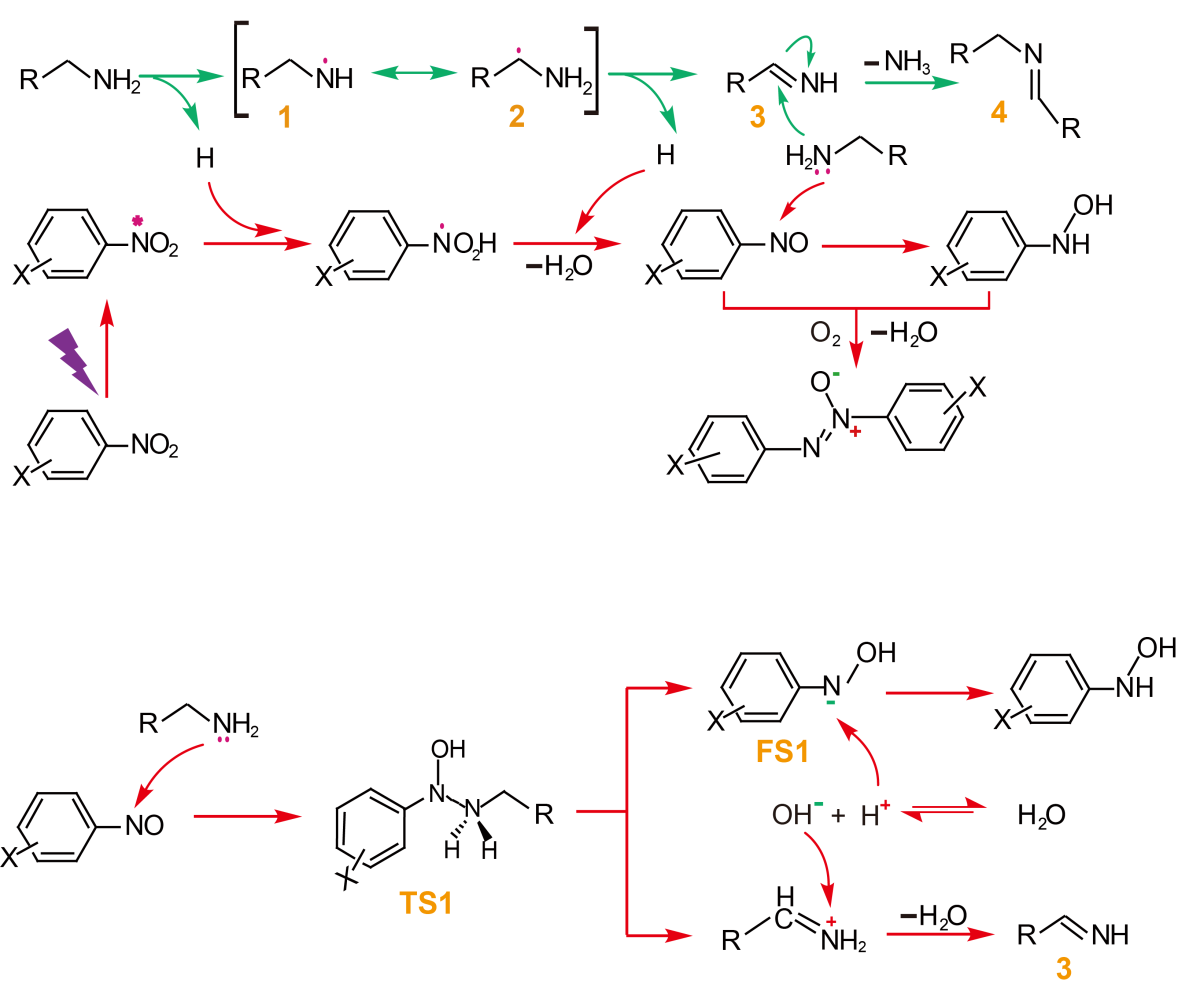


**Figure S8.** The probable mechanism profiles of the generation process of PHA and propanimine.


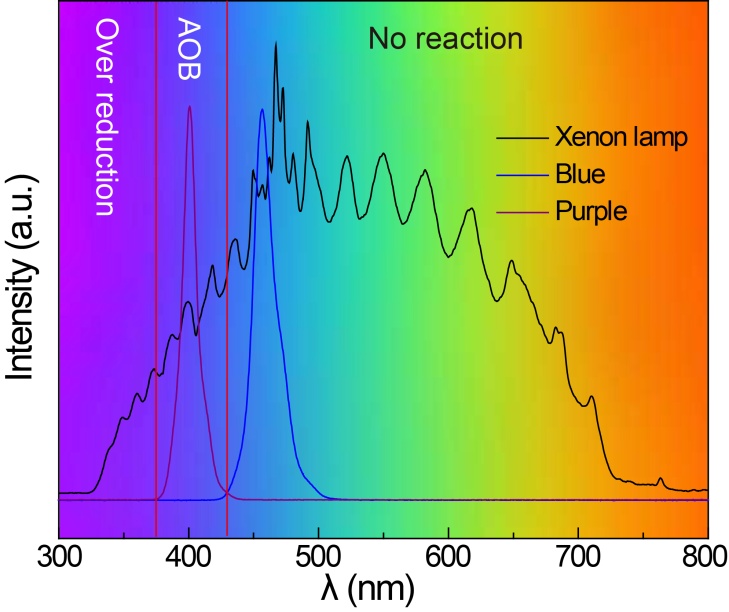


**Figure S9.** The spectrum of Xenon lamp, LED purple and blue light.

We can tune the selectivity of the reaction by change the light source. As shown in Figure S9, no reaction is occurred by using LED blue light or longer band light. However, using LED purple light can get the AOB product, once the shortwave light is introduced for this reaction the over reduction product such as azobenzene and aniline can be detected.

References

1. Frisch, M. J. T., G. W.; Schlegel, H. B.; Scuseria, G. E.; Robb, M. A.; Cheeseman, J. R.; Scalmani, G.; Barone, V.; Mennucci, B.; Petersson, G. A.; Nakatsuji, H.; Caricato, M.; Li, X.; Hratchian, H. P.; Izmaylov, A. F.; Bloino, J.; Zheng, G.; Sonnenberg, J. L.; Hada, M.; Ehara, M.; Toyota, K.; Fukuda, R.; Hasegawa, J.; Ishida, M.; Nakajima, T.; Honda, Y.; Kitao, O.; Nakai, H.; Vreven, T.; Montgomery, Jr., J. A.; Peralta, J. E.; Ogliaro, F.; Bearpark, M.; Heyd, J. J.; Brothers, E.; Kudin, K. N.; Staroverov, V. N.; Kobayashi, R.; Normand, J.; Raghavachari, K.; Rendell, A.; Burant, J. C.; Iyengar, S. S.; Tomasi, J.; Cossi, M.; Rega, N.; Millam, J. M.; Klene, M.; Knox, J. E.; Cross, J. B.; Bakken, V.; Adamo, C.; Jaramillo, J.; Gomperts, R.; Stratmann, R. E.; Yazyev, O.; Austin, A. J.; Cammi, R.; Pomelli, C.; Ochterski, J. W.; Martin, R. L.; Morokuma, K.; Zakrzewski, V. G.; Voth, G. A.; Salvador, P.; Dannenberg, J. J.; Dapprich, S.; Daniels, A. D.; Farkas, Ö.; Foresman, J. B.; Ortiz, J. V.; Cioslowski, J.; Fox, D. J. Gaussian 09 Revision A.02 ed. Wallingford CT: Gaussian, Inc.; 2009.

2. Ochterski, J. W. Thermochemistry in Gaussian2000.

3. Lamson, D. W., Sciarro, R., Hryb, D., Hutchins, R. O. Oxidaton of benzylamines with nitrosobenzene. *The Journal of Organic Chemistry* **38**, 1952-1954(1973).

4. Wu, Y. M., Ho, L. Y., Cheng, C. H. (Phenylazo) Alkanes from reaction of nitrosobenzene with alkylamines. *Journal of Organic Chemistry* **50**, 392-394(1985).
